# The Impact of Restricting Over-the-Counter Sales of Antimicrobial Drugs: Preliminary Analysis of National Data

**DOI:** 10.1097/MD.0000000000001605

**Published:** 2015-09-25

**Authors:** Maria Luísa Moura, Icaro Boszczowski, Naíma Mortari, Lígia Vizeu Barrozo, Francisco Chiaravalloti Neto, Renata Desordi Lobo, Antonio Carlos Pedroso de Lima, Anna S. Levin

**Affiliations:** From the Infection Control Department, Hospital das Clínicas (MLM, IB, NM, RDL, ASL); Department of Geography, Faculdade de Filosofia, Letras e Ciências Humanas (LVB); Department of Epidemiology, Faculdade de Saúde Pública (FCN); Department of Statistics, Institute of Mathematics and Statistics, (ACPDL); and Department of Infectious Diseases and LIM 54, Faculdade de Medicina, University of São Paulo, São Paulo, Brazil (ASL).

## Abstract

To describe the nationwide impact of a restrictive law on over-the-counter sales of antimicrobial drugs, implemented in Brazil in November 2010.

Approximately 75% of the population receives healthcare from the public health system and receives free-of-charge medication if prescribed. Total sales in private pharmacies as compared with other channels of sales of oral antibiotics were evaluated in this observational study before and after the law (2008–2012). Defined daily dose per 1000 inhabitants per day (DDD/TID) was used as standard unit.

In private pharmacies the effect of the restrictive law was statistically significant (*P* < 0.001) with an estimated decrease in DDD/TID of 1.87 (s.e. =  0.18). In addition, the trend of DDD/TID before the restrictive law was greater than after the intervention (*P* < 0.001). Before November 2010, the slope for the trend line was estimated as 0.08 (s.e. = 0.01) whereas after the law, the estimated slope was 0.03 (s.e. = 0.01). As for the nonprivate channels, no difference in sales was observed (*P* = 0.643). The impact in the South and Southeast (more developed) regions was higher than in the North, Northeast, and Mid-West. The state capitals had a 19% decrease, compared with 0.8% increase in the rest of the states.

Before the law, the sales of antimicrobial drugs were steadily increasing. From November 2010, with the restrictive law, there was an abrupt drop in sales followed by an increase albeit at a significantly lower rate. The impact was higher in regions with better socio-economic status.

## INTRODUCTION

The spread of antimicrobial resistance is a major threat to public health and is considered by the World Health Organization to be a global concern that requires urgent action. Misuse and over-exposure to antibiotics is considered a major factor in accelerating the emergence of multidrug-resistant organisms.^1^ These pathogens, although usually demonstrated in the hospital environment, are increasingly prevalent in community-acquired infections.^2–7^ Resistance in *Mycobacterium tuberculosis,* and *Neisseria gonorrhea*, and to antimalarial drugs such as chloroquine and sulfadoxine-pyrimethamine, is widespread in most countries.^8^ The World Health Assembly Resolution of 1998 urged Member States to develop measures to encourage appropriate and cost-effective use of antimicrobials.^9^

The misuse of antibiotics for viral infections and the excessive use of broad-spectrum antibiotics have been well documented.^10–13^ One approach toward reducing the incidence of infections due to antibiotic-resistant organisms is to reduce the inappropriate use of these drugs in both the hospital and community settings.^11^ To this end, public policies that restrict the consumption of antimicrobials in the community are generally recommended; however data evaluating their impact are scarce.

In Brazil, over-the counter sales were common until November 2010 when a national restrictive law requiring medical prescription for the sale was implemented.^14^ This approach had been applied in other Latin America countries, with controversial results as to its impact. The objective of this study was to describe the impact of the restrictive law on the prescription of antimicrobials for outpatients on the sales of these drugs in Brazil.

## METHODS

### Study Area

Brazil is a country with approximately 200,000,000 inhabitants, according to the Brazilian Institute of Geography and Statistics (Instituto Brasileiro de Geografia e Estatística (IBGE))^15^ and is divided into 5 macro regions: Mid-West, Northeast, North, Southeast, and South. These 5 regions are divided into 26 states, which are split into municipalities (over 5500). The capital, Brasília, is located in the Mid-West region. The country has a Unified National Health System (Sistema Único de Saúde (SUS)) started in 1990. SUS, in theory, provides coverage to the entire population of the country; thus healthcare is universal and free for the user. Healthcare management in the SUS is decentralized.^16^ Most of primary care is the responsibility of the municipality. Hospitals of the SUS are either public or private with public funding. When a drug is prescribed by a doctor within the SUS, the patient can receive it free of charge in the healthcare system (primary care unit, outpatient clinic, or hospital) or in a public pharmacy. This dispensation requires the patient to provide a prescription. Patients with prescriptions issued outside the SUS are obliged to purchase their drug in private pharmacies. In 2008, 26% of the population had a private health plan or insurance.^17^ In private pharmacies, many drugs, including antimicrobials, could have been bought over-the-counter. This ended with the restrictive law in November 2010.

### Antimicrobial Consumption

Data on sales of oral antimicrobial drugs were obtained from audits performed by Intercontinental Medical Statistics Health Brazil (IMS Health Brazil), an international company for the pharmaceutical industry marketing research. This company has information about 98% of the sales of antimicrobials in the country.

We evaluated the data available on the sales to private pharmacies and assumed that all antimicrobials were consumed within the month of purchase. The formulations containing amoxicillin conjugated with other drugs (eg, clavulanic acid) were grouped with formulations containing amoxicillin only.

Defined daily dose per 1000 inhabitants per day (DDD/TID) was used as the standard unit, according to the Anatomical Therapeutic Chemical classification system.^18^ This unit reflects the defined daily doses consumed per 1000 inhabitants in 1 day and was calculated as follows:

*Monthly rate*: DDD/1000 inhabitants-day = [quantity of antibiotic in a given month (g) × 1000]/[30 days × DDD for that drug × population].

*Yearly rate*: DDD/1000 inhabitants-day = [quantity of antibiotic in a given year (g) × 1000]/[365 days × DDD for that drug × population].

For DDD/TID, computed on a monthly basis, a segmented regression analysis was considered to assess the effect of the restrictive law on sales to private pharmacies, as well as to other channels of sales. In order to take into account seasonality as well as possible autocorrelation, 12 dummy variables were included in the model, 1 for each month of the year. Durbin–Watson test, correlation, and autocorrelation plots were used to evaluate the usual assumptions of the model. Time was considered through an index variable, varying from 0 to 60, with a change-point around November, 2010. The intercepts and slopes associated with trend lines were considered in evaluating the effect of the restrictive law on sales. Differences in sales around the change-point (November 2010) and trends before and after the restrictive law were assessed based on the Wald test.

We also evaluated the yearly consumptions of antibiotic therapeutic subgroups – penicillins, cephalosporins, quinolones, macrolides, sulphonamides, and others.

Yearly regional and state consumption were calculated for each antibiotic. We also evaluated the consumption in large urban areas (state capitals) as well as the sales of each state excluding the metropolitan areas of the state capitals. The population data for each year were obtained from IBGE.^15^

Sales to nonprivate pharmacies (mainly those in which SUS healthcare is provided) were evaluated monthly basis only. Due to problems with these data, the evaluation was started in July 2008. This “nonprivate” category (denoted “other channels of sales”) included primary care units, outpatient clinics, SUS-affiliated hospitals, and public pharmacies.

This study was part of a more ample study approved by the Ethics Committee (number 443/11).

## RESULTS

**FIGURE 1:**
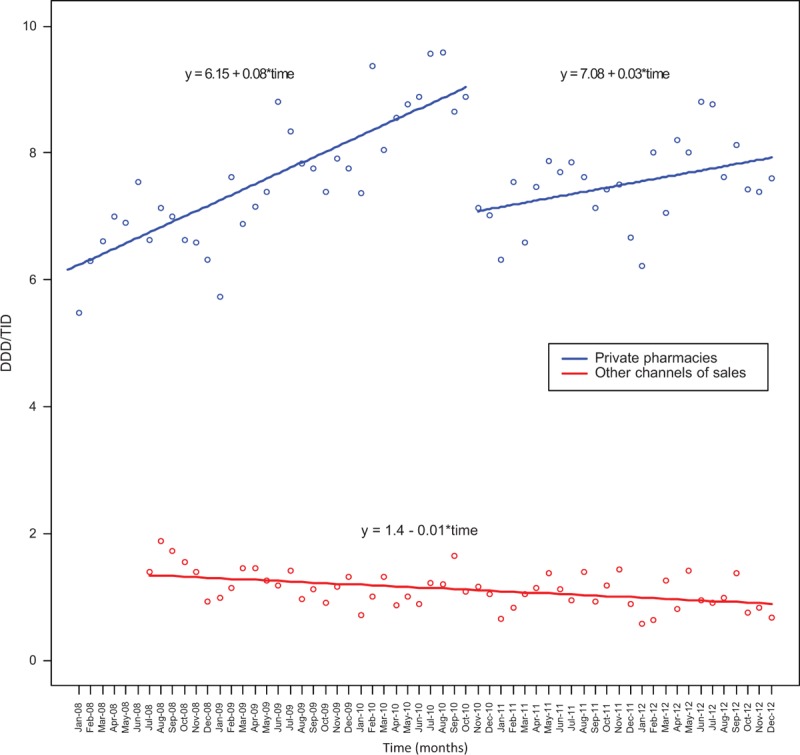
Monthly rates of sales of oral antimicrobial drugs in Brazil (2008–2012). In November 2010, a national law, restricting over-the-counter sales of antimicrobial drugs, was implemented. (DDD/TID = daily defined doses per 1000 inhabitants per day).

The sales for the entire period are shown in Figure 1 (dots) accompanied by trend lines obtained by fitting the data considering the segmented regression model, for private pharmacies (in blue) and other channels of sales (in red). For private pharmacies the effect of the restrictive law was statistically significant (*P* < 0.001) with an estimated decrease in DDD/TID of 1.87 (s.e. = 0.18). In addition, the trend of DDD/TID before the restrictive law was greater than after the intervention (*P* < 0.001). Before November 2010, the slope for the trend line was estimated as 0.08 (s.e. = 0.01) whereas after November 2010, the estimated slope was 0.03 (s.e. = 0.01). As for sales related to other channels of sales, no difference was observed because of the restrictive law (*P* = 0.643).

The 11 most sold antibiotics were amoxicillin, cotrimoxazole, azithromycin, ciprofloxacin, cephalexin, levofloxacin, norfloxacin, nitrofurantoin, penicillin, moxifloxacin, and doxycycline (Table 1). They represented 96% of the total oral antibiotic sales in the study period.

**TABLE 1:**
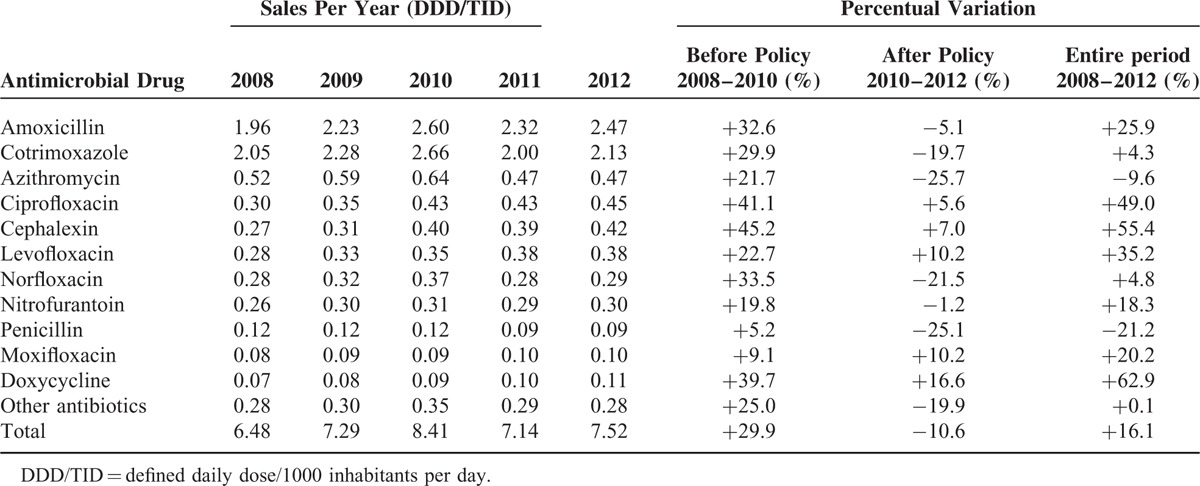
Sales of Oral Antimicrobial Drugs in Brazil From 2008 to 2012 and Variation Comparing Periods Before and After Restriction Policy

**FIGURE 2:**
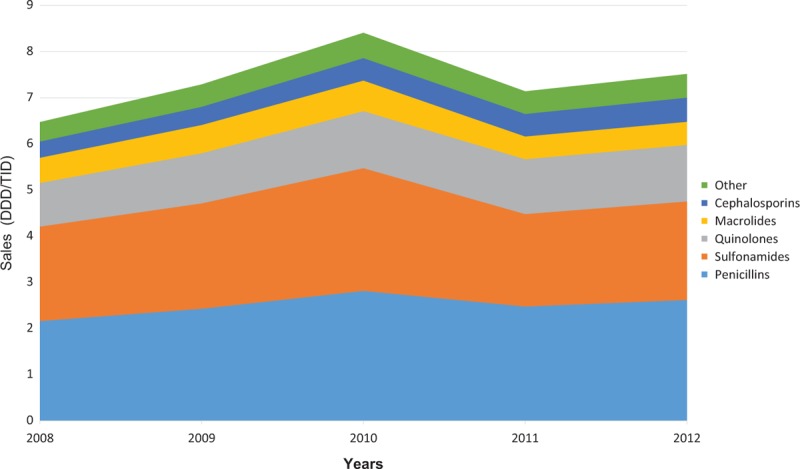
Oral antimicrobial sales in Brazil by therapeutic subgroup, 2008–2012. (DDD/TID = defined daily dose per 1000 inhabitants-day).

Analyzing the sales for each therapeutic subgroup, we observed that penicillins were the most sold antibiotics, followed by sulfonamides (cotrimoxazole), quinolones, macrolides, and cephalosporins. After the implementation of the restrictive law, there was a proportional decrease in the sales of macrolides and sulfonamides, while the sales of quinolones continued to rise (Figure 2).

Sales increased from 2008 to 2010 for the 11 drugs. After the new law, reduction was approximately 15% for cotrimoxazole, azithromycin, norfloxacin, and penicillin. Amoxicillin and nitrofurantoin presented a less than 15% reduction. For the remaining antimicrobials, although there was an increase in sales, growth seemed to be lower when compared with the period before regulation. The exception was moxifloxacin, which increased consistently (Table 1).

**FIGURE 3:**
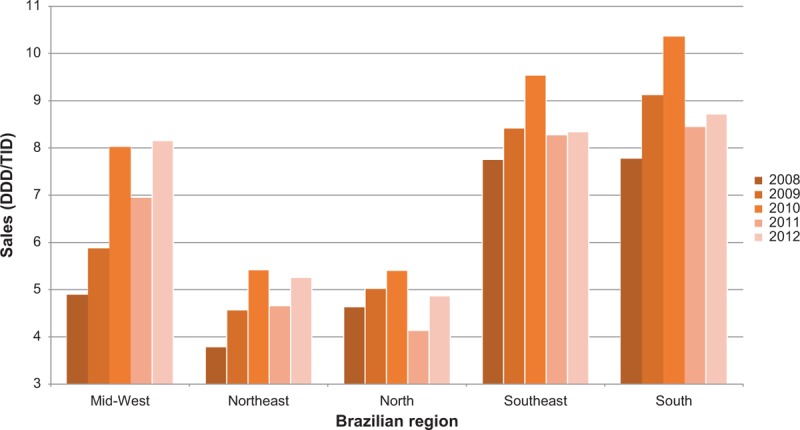
Sales of the eleven most sold oral antibiotics in Brazil according to the region (DDD/TID = defined daily dose per 1000 inhabitants-day) from 2008 to 2012.

Comparing the 5 great Brazilian regions, we observed a higher impact in the more developed regions: South and Southeast (reduction of 13% and 16%, respectively), compared with the North, Northeast where there was a smaller impact (reduction of 7% and 3%, respectively). There was no impact in the Mid-West (variation of +2%) (Figure 3). Only penicillin, azithromycin, and norfloxacin presented a decrease in all 5 regions. Cotrimoxazole presented a reduction superior to 20% in all regions except the Mid-West. In contrast, levofloxacin, moxifloxacin, and doxycycline had a positive variation in sales in all regions after the restrictive law. Even for antibiotics that presented increased sales after regulation, this rise was less than 10% for all in Southeast and South. The exception was doxycycline (Table 2).

**TABLE 2:**
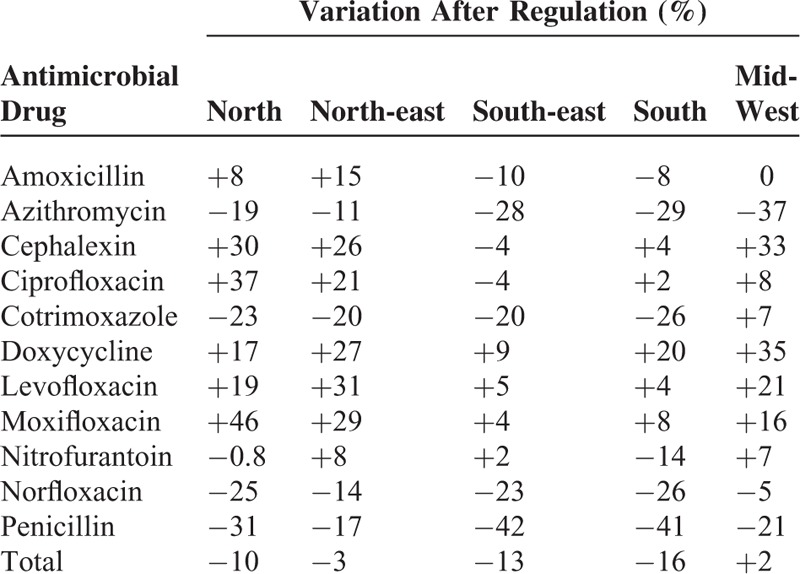
Variation in Sales of the 11 Most Sold Oral Antibimicrobial Drugs in Brazil After the Restrictive Policy, According to Region

**FIGURE 4:**
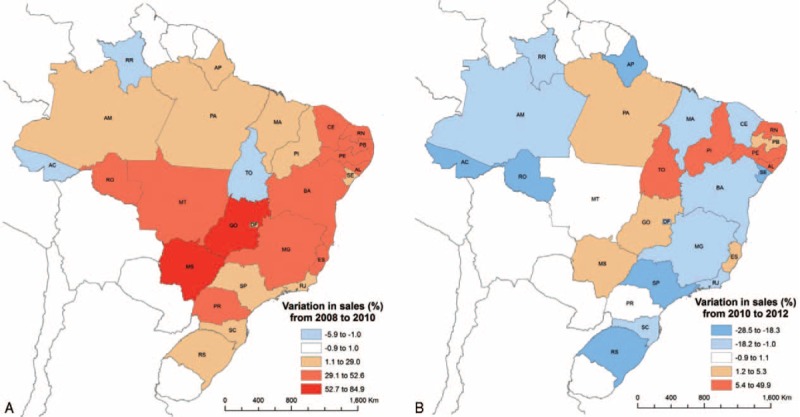
Variation in sales of the eleven most sold oral antibiotics in Brazil by State, before (A) and after (B) the restrictive law.

**FIGURE 5:**
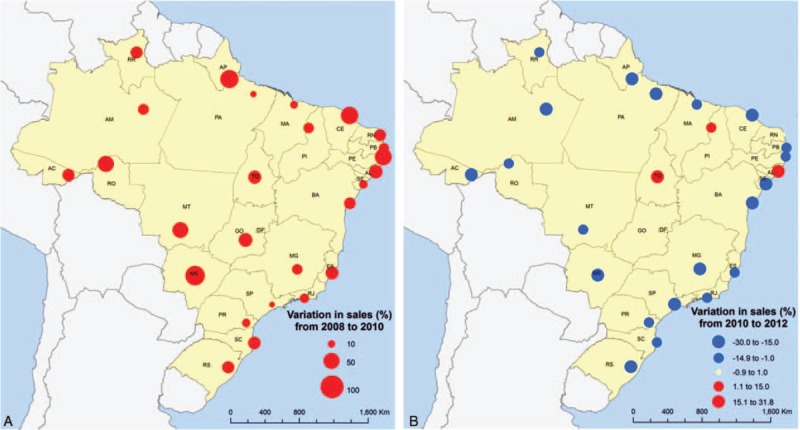
Variation in sales of the eleven most sold oral antibiotics in Brazil in State Capitals, before (A) and after (B) the restrictive law.

**FIGURE 6:**
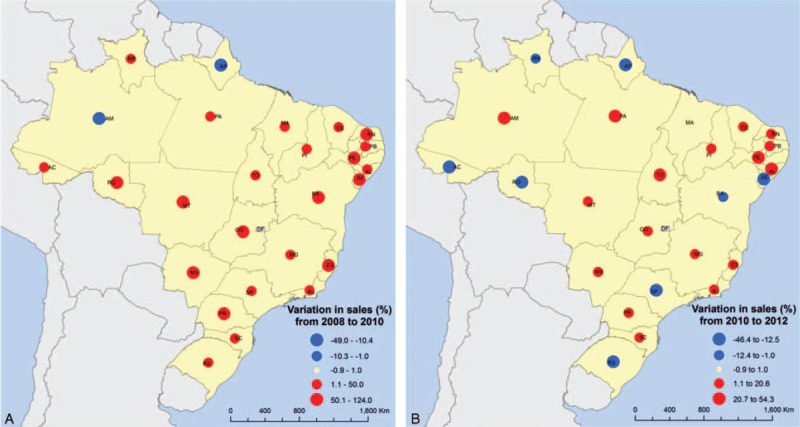
Variation in sales of the 11 most sold oral antibiotics in Brazil in the areas excluding the capitals in each state, before (A) and after (B) the restrictive law.

Sixteen of 26 states had a decrease in sales after the law. Among the 10 states in which sales increased after 2010, 8 had a smaller than 10% increase and 2 presented an increase greater than 20% (Figure 4). All but 3 of the state capitals had a reduction of sales from 2010 to 2012, and this reduction was >20% in 20 of 26 state capitals. In contrast, excluding the capitals, there was a positive variation between 2010 and 2012 (+0.8%). Only 4 of these areas had a more than a 20% reduction in the period after the law (Figures 5 and 6).

## DISCUSSION

Our study showed that a national law that banned over-the-counter sales of antimicrobial drugs, in place since November 2010, had an impact on sales in private pharmacies but did not affect other sectors such as healthcare provided by the Brazilian unified health system SUS.

Before the law, sales of antimicrobial drugs were steadily increasing. With the restrictive law, in November 2010, there was an abrupt drop in sales followed by an increase at a significantly lower rate.

In Brazil, 26% of the population is assisted by the private sector.^19^ Historically, there was a liberal policy for antimicrobial use in the community which meant that although, there was a formal recommendation for selling antimicrobial drugs with a medical prescription, no inspection or control was applied in private pharmacies. On the other hand, in public healthcare facilities and pharmacies, a medical prescription has always been necessary for free-of-charge drug dispensation. The law therefore would not be expected to have an effect in this sector. In view of this consideration, we decided to evaluate private pharmacies separately. Our results are different from a similar study done Brazil^20^ in which the overall sales of antibiotics increased despite the restriction on over-the-counter sales.

Over-the-counter restriction of sales of antibiotics has been implemented in other Latin American countries.^21^ In Chile, there was a decrease of 5.5 DDD/TID in consumption after restriction in 1999,^22^ but this impact was not sustained after 2002.^23^ In Colombia, the measure was restricted to the capital and implemented in 2005, causing a decrease of 1 DDD/TID, which was considered a small effect.^24^ In 2006 in Venezuela, the regulation was applied only to certain classes of antibiotics and had no significant effect on the consumption.^25^ In Mexico, the restriction was implemented in 2010, with reduction of 3 DDD/TID (29% decrease); however, there had already been a trend toward decrease of consumption before the regulation.^20^

When analyzing the impact of the restriction in each of the 5 macro regions of Brazil, we observed a decrease in all during the year following the implementation (2011). This was sustained only in the South and Southeast. The other regions returned to levels close to the preintervention period. The South and Southeast are the most developed regions in terms of socioeconomic conditions and have better access to healthcare systems (measured by illiteracy rate, number of health professionals, and number of health establishments per inhabitant, human development index).^26,27^ We hypothesize that these regions are more frequently inspected by the regulatory organisms than the other regions. Moreover, people living in poorer areas are more likely to be assisted by the public health system and less by the private sector and this may partially explain the lesser effect of the law. The same reasoning may explain the greater impact observed in capitals and metropolitan regions. It is possible that in small towns, sales still occur without strict control.

Azithromycin, penicillin, norfloxacin, and cotrimoxazole presented a decrease in sales in all regions, (with the exception of cotrimoxazole in the Mid-West). These are common medications used for sore throat, respiratory illness, and urinary tract infections and are well known to the lay person. We hypothesized that they may have been sold without a medical prescription despite the law. Stratchounski et al demonstrated that the major indications of automedication with antibiotics in big cities in Russia were acute viral respiratory tract infections, cough, intestinal disorders, fever, and sore throat.^28^ To our knowledge, there are no similar studies in Brazil, so it is not sure whether drugs are used through automedication or whether they are indicated by the pharmacists. Notably, there is a shortage of physicians in many small towns and even in peripheral areas of metropolitan regions, which might lead people to look for healthcare assistance in the pharmacies. In fact, this paucity of medical professionals in certain regions is the object of a nationwide program instituted in 2013 by the Brazilian Federal government to better medical assistance in these areas.^29^

On the other hand, doxycycline, moxifloxacin, and levofloxacin showed a consistent increase in sales during the period after the law. This is difficult to explain. Doxycycline is an antimicrobial mainly prescribed for the treatment of sexually transmitted diseases.^30^ We do not have data on the incidence of these infections. However data on syphilis during pregnancy, which increased from 2.3/1000 babies born in 2008 to 5/1000 babies born in 2011,^31^ suggest that sexually transmitted diseases may be on the rise. This may explain the increase in the sales of doxycycline. Moxifloxacin and levofloxacin are relatively new drugs that are commonly prescribed by physicians in emergency services. Due to the convenience of once-daily dosing and good spectrum to treat the main microorganisms that cause upper and lower respiratory tract infections, physician preference may explain this increase during the study period.

It is necessary to clarify which are the factors that influence medical prescription in Brazil. Balabanova et al, in a cross-sectional survey in Russia, demonstrated that 80% of the doctors made extensive use of pharmaceutical company information to prescribe antimicrobials for respiratory diseases.^32^ It could be interesting to investigate to what extent pharmaceutical companies, medical societies, government educational programs, and formal school education influence medical prescribing criteria. It is important to highlight that the restrictive law was implemented in Brazil without any educational interventions. Certainly, other measures are necessary to control the overuse of antimicrobials in Brazil, including medical education.

Our study has limitations. First, our data on sales of the pharmaceutical companies do not guarantee that the drugs bought were effectively dispensed to patients and used by them. Also, when calculating monthly DDD/TID rates we had to assume that the population was stable over the entire year, as we only had yearly population data. Furthermore, factors other than the restriction of over-the-counter sales may have had an influence, for example, changes in the economy, demographic factors, and the influence of the pharmaceutical industry. These considerations require further studies.

In conclusion, there was an important drop in antimicrobial sales in Brazil after a national restrictive law. This impact was higher for the large urban areas and regions with better socio-economic status. Further investigation is necessary to clarify determinant factors for these regional differences.
